# MTHFR 677C>T Polymorphism and the Risk of Breast Cancer: Evidence from an Original Study and Pooled Data for 28031 Cases and 31880 Controls

**DOI:** 10.1371/journal.pone.0120654

**Published:** 2015-03-24

**Authors:** Singh Pooja, Justin Carlus, Deepa Sekhar, Amirtharaj Francis, Nishi Gupta, Rituraj Konwar, Sandeep Kumar, Surender Kumar, Kumarasamy Thangaraj, Singh Rajender

**Affiliations:** 1 Division of Endocrinology, Central Drug Research Institute, Lucknow, India; 2 Department of Pathology, King George’s Medical University, Lucknow, India; 3 Centre for Genetics and Inherited Diseases (CGID), Taibah University, Al- Madinah, Kingdom of Saudi Arabia; 4 Centre for Cellular and Molecular Biology, Uppal Road, Hyderabad, India; 5 All India Institute of Medical Sciences, Bhopal, India; 6 Department of Surgery, King George’s Medical University, Lucknow, India; National Cancer Center, JAPAN

## Abstract

**Background:**

Methylenetetrahydrofolate reductase (MTHFR) acts at an important metabolic point in the regulation of cellular methylation reaction. It assists in the conversion of 5, 10-methylenetetrahydrofolate to 5-methyltetrahydrofolate. The latter aids in remethylation of homocysteine to *de novo* methionine that is required for DNA synthesis. The objective of this study was to examine the effect of MTHFR 677 C>T polymorphism on the risk of breast cancer in the Indian sub-continent.

**Methods and Results:**

We genotyped 677 C>T locus in 1096 individuals that were classified into cases (N=588) and controls (N=508). Genotype data were analyzed using chi-square test. No significant difference was observed in the distribution of genotypes between cases and controls in north Indian (P = 0.932), south Indian (P = 0.865), and pooled data (P = 0.680). To develop a consensus regarding the impact of 677C>T polymorphism on breast cancer risk, we also conducted a meta-analysis on 28031 cases and 31880 controls that were pooled from sixty one studies. The overall summary estimate upon meta-analysis suggested no significant correlation between the 677C>T substitution and breast cancer in the dominant model (Fixed effect model: OR = 0.97, P=0.072, Random effects model: OR = 0.96, P = 0.084) or the recessive model (Fixed effect model: OR = 1.05, P = 0.089; Random effects model: OR= 1.08, P= 0.067).

**Conclusion:**

677 C>T substitution does not affect breast cancer risk in the Indo-European and Dravidian populations of India. Analysis on pooled data further ruled out association between the 677 C>T polymorphism and breast cancer. Therefore, 677 C>T substitution does not appear to influence the risk of breast cancer.

## Introduction

Breast cancer has become the most common cancer among women with a consistent increase in frequency. The genetic damage caused by endogenous metabolites and exogenous risks might explain nature of the disease [1]. The exact causes of breast cancer are unknown, but a number of factors may contribute to the development of the disease, such as age of menarche and menopause, diet and exposure to high estrogen levels [2]. The etiology of the disease links to various genetic and epigenetic processes, including DNA synthesis, methylation, and repair [3]. Two important mechanisms that might lead to the risk of malignancy are: 1) DNA hypomethylation and activation of proto-oncogenes. 2) Misincorporation of uracil during DNA synthesis, leading to catastrophic DNA repair and chromosome damage [4]. Folate, an important dietary component, is found in legumes, green leafy vegetables, and liver, and the role of this B vitamin involves the transmission of one carbon group to carry out necessary biological reactions [5]. Deficiency of folate caused by low dietary intake, diminished metabolism or no auxiliary intake may result in breakage of DNA strands, increased rate of mutagenesis and changes in the DNA methylation patterns, ultimately affecting the expression of a number of genes [6, 7]. Lack of folate is believed to affect the risk of cancer through the processes described above.

The methylenetetrahydrofolate reductase (MTHFR) gene is mapped to chromosome 1p36.3 and consists of a 2 kbp coding region divided into eleven exons [8]. It plays an important role in the regulation of cellular methylation by assisting the conversion of 5, 10-methylenetetrahydrofolate to 5-methyltetrahydrofolate [9]. The latter aids in the remethylation of homocysteine to de novo methionine [10], which serves as a precursor for the S-adenosylmethionine, a universal methyl donor for methylation reactions [11]. It also functions as a coenzyme in purine and thymidylate synthesis. Two functional polymorphisms in the *MTHFR* gene, 677C>T (ala→val) and 1298A>C (glu→ala), have a profound effect on the activity of enzyme, producing more labile forms with reduced activity [12]. The 677C>T is a common SNP, which converts an alanine to valine at codon 225 of the folate binding site of methylenetetrahydrofolate reductase [13]. The enzyme with homozygous and heterozygous substitutions exhibit 30% and 65% activity, respectively, in comparison to the wild type [9]. Since low dietary folate intake is correlated with an increase in the rate of breast cancer, MTHFR 677C>T may affect breast cancer risk by negatively modifying folate levels [14]. In this case-control study, we investigated if MTHFR 677 C>T polymorphism affects breast cancer risk. A few meta-analyses have reported an association between 677 C>T polymorphism and breast cancer risk [10, 15, 16, 17, 18]; however, none of these has addressed the issue using a meticulous plan taking into consideration sensitivity analysis that may significantly affect the outcome. Repeated meta-analysis using similar strategy does not add new information to the literature. The principal of statistics that odd observations should not be favoured unless the evidence is very compelling, propelled us to undertake a stringent statistical approach and sensitivity analysis to critically look into the relationship between the 677 C>T polymorphism and breast cancer risk.

## Materials and Methods

### Case-control study

#### Subjects

The study and the protocol for sample collection were approved by the Institutional Human Ethics Committee of the King George’s Medical University (KGMU), Lucknow, India. Informed written consents of the participants were obtained, and no minor subjects were enrolled in the study. The consent procedure was approved along with the study protocol by the Institutional Ethics Committee. The study included two ethnically different case-control groups from the Indian sub-continent. A pre-defined set of recruitment/exclusion protocol was followed for both groups.

The north Indian group consisted of breast cancer cases (N = 331) and controls (N = 181) of the Indo-European linguistic group from Uttar Pradesh. The subjects were recruited from the Department of Surgery and Oncology, KGMU, Lucknow, India. The age of the patients varied from 22 to 90 years with a mean age of 42.11 years (SD 14.21). In this group, 191 cases were pre-menopausal and 140 were post-menopausal. One hundred fifty-five patients had cancer in the right breast, 160 patients in the left breast, and only 16 patients in both the breasts. The size of tumours varied from a minimum of 3 cm^3^ to a maximum of 1150 cm^3^ with a mean value of 125.93 cm^3^ (SD, 324.43). The staging of tumours was done according to the TNM classification. Three patients (0.91%) were in stage I, 123 patients (37.16%) were in stage II, 159 patients (48.03%) were in stage III, and 46 patients (13.89%) were in stage IV. Grading of tumours was done according to the Bloom-Richardson grading system, where the tumor grade was decided according to the overall score that a tumor got upon analyzing for the degree of tumor tubule formation, tumor mitotic activity, and tumor nuclear grade. Healthy controls were recruited from the out-patient department and staff members of the Department of Surgery and Oncology. The controls had no family history of breast cancer and all had undergone a recent mammogram confirming that there was no detectable breast cancer at the time of sampling. Age of the controls ranged from 28 to 70 years with a mean age of 40 years (SD, 12.40). It was ensured that patients and controls were enrolled from the populations of same ethnicity.

The south Indian group consisted of patients (N = 257) and controls (N = 327) of the Dravidian linguistic group. The age of the patients ranged from 24 to 82 years with a mean age of 48.32 years (SD 12.25). One hundred and two cases were pre-menopausal and 155 were post-menopausal. One hundred and nineteen patients had cancer in the right breast, 128 patients in the left breast, and 10 in both breasts. Size of the tumours varied from a minimum of 6 cm^3^ to a maximum of 1310 cm^3^ with a mean value of 131.23 cm^3^ (SD, 347.12). The staging of the tumours was done according to the TNM classification. Two patients (0.78%) were found to be in stage I, 103 patients (40.07%) were in stage II, 142 patients (55.25%) were in stage III, and 10 patients (3.89%) were in stage IV. Ethnically matched controls were recruited from the out-patient department and staff members who had no family history of breast cancer. The controls had undergone a recent mammogram confirming absence of breast cancer at the time of sampling. Age of the controls ranged from 32 to 70 years with a mean age of 48 years (SD 12.37). Further details of the patients and controls are presented in Table 1.

**Table 1:** Descriptive data of cases and controls.

	North Indian		South Indian	
Variables	Cases	Controls	Cases	Controls
Age (mean±SD)	42.11±14.21	40±12.40	48.32±12.25	48 ± 12.37
BMI (Kg/m^2^)	22.41+5.87	23.21 ± 5.81	22.19+5.21	22.36± 5.21
Age at menarche (years, mean ± SD)	13.76 ± 1.72	13.54 ± 1.78	13.91 ± 1.18	13.52 ± 1.29
Age at diagnosis for cases or at interview for controls				
≤ 30 years	54	16	4	34
31–45 years	134	87	81	114
46–60 years	97	53	117	119
61–75 years	36	17	50	50
76–90 years	10	8	5	10
Family history				
Positive	23	0	16	0
Negative	308	181	241	327
Tobacco chewing/smoking habit				
Yes	20	10	31	12
No	311	171	226	315

#### Isolation of genomic DNA

DNA from the peripheral blood samples was extracted using the phenol-chloroform-isoamyl method. The 677C>T polymorphism was genotyped using the PCR-RFLP method. Primers in the vicinity of polymorphic site were designed with the GENETOOL software. PCR reactions of 10μl volume were performed in thin walled PCR tubes consisting of 1.0μl of PCR buffer (10X), 1.0μl of dNTPs (10mM), 2.0μl of each of the forward (5’CATCCCTATTGGCAGGTTACCC3’) and reverse (5’GGGAAGAACTCAGCGAACTCAG3’) primers, 0.2 μl of Taq DNA polymerase enzyme (Applied Biosystems), and 40ng of genomic DNA. PCR was carried out in ABI Veriti thermal cycler (Applied Biosystems, USA). PCR conditions consisted of: denaturation at 95°C for 5 minutes, followed by 35 cycles of denaturation at 95°C for 30 seconds, annealing at 66°C for 30 seconds, polymerization at 72°C for 20 seconds, and a final stage polymerization at 72°C for 7 minutes. The products were digested with *HinfI* in a total volume of 10 μl, and the fragments were separated on a 3.0% agarose gel. The C>T substitution created a restriction site for *HinfI* that produced fragments of 225bp and 93bp upon restriction digestion. Representative samples of each genotype were sequenced by direct DNA sequencing to confirm genotyping results produced by RFLP.

#### Statistical analysis

Genotype data for control population was studied for fitness in the Hardy Weinberg Equilibrium (HWE). For this purpose, data was analyzed using calculator available at http://ihg.gsf.de/cgi-bin/hw/hwa1.pl. Chi-square analysis was done to compare the genotype data between cases and controls. Data were analyzed using the online statistical tool available at Vassar Stats online calculator http://faculty.vassar.edu/lowry/VassarStats.html. Significance was present if p values were less than 0.05.

### Meta-analysis

677C>T in breast cancer has been studied in several ethnic groups, making it valuable to conduct a meta-analysis. We have used the Comprehensive Meta Analysis software (version 2) for this purpose.

#### Identification of studies

A thorough electronic search of the published literature was done in ‘Google Scholar (scholar.google.co.in)’, ‘Pubmed (http://www.ncbi.nlm.nih.gov/pubmed/)’ and ‘Sciencedirect (www.sciencedirect.com)’ databases up to August 2014 as the publication date, using the following keywords: breast cancer, *MTHFR* 677C>T polymorphism, folate metabolism, and breast cancer in different combinations. Detailed information regarding data presentation, design and purpose of the study, method of genotyping, and inclusion and exclusion criteria of the subjects were collected. The authors were contacted by e-mail when published information was inadequate for inclusion in meta-analysis. Meta analyses published to date suggest a significant correlation of 677 C>T polymorphism with breast cancer. Most of these pooled data analysis lack quality control and sensitivity analysis. We have undertaken a meta-analysis on published data in order to look into the association between 677 C>T polymorphism and breast cancer risk.

#### Inclusion and exclusion criteria

The inclusion criteria comprised of the following: i) Each study was an independent case-control study ii) The statistical methods and purpose of all the studies were similar iii) The given information was enough to calculate the odds ratio iv) SNP genotyping was done using standard genotyping techniques v) Patients were recruited in accordance with the standard diagnostic parameters. The exclusion criteria included: the raw data were unavailable in the article or the authors did not respond after three requests by e-mail.

#### Data extraction and statistical approach

The genotype data for *MTHFR* 677C>T polymorphism in relation to breast cancer risk were collected. The required information such as the first author, ethnicity of the study population, publication year, number of cases and controls, and the frequency of genotypes were gathered.

#### Statistical analysis

Meta-analysis was conducted using the Comprehensive Meta-Analysis (CMA) software (version 2), which allows data entry in various formats. The ‘effect size’ was considered an important criterion to design and interpret the results of meta-analysis that compared CC versus CT+TT genotypes in the dominant model and CC+CT versus TT in the recessive model. We chose the effect size calculated in the form of ‘odds ratio’ for data interpretation. To calculate heterogeneity quantitatively, Thompson and Higgins classification index, I^2^, was taken into account, where a proposed range of 25%, 50%, and 75% is set that corresponds to low, medium, and high magnitudes of heterogeneity [19]. In the absence of heterogeneity, fixed effect model using the Mantel-Haenzel method was used for pooled data analysis, else the random effects model using the Der Simonian and Laird method was applied [19,20]. High resolution plot (Forest plot) was produced to estimate the pooled odds ratio and p value. The p values less than 0.05 were considered statistically significant. The results and the robustness of the methodology were checked by sensitivity analysis, whereby, studies using small sample size (<100) in either of the study groups were excluded followed by re-analysis of the data. Sensitivity analysis aims at identifying the studies that are sensitive enough to significantly bias the results of pooled analysis. A study may be sensitive due to a variety of reasons, such as the use of small sample size, large variation in the number of cases and controls analyzed and poor methods, of which the use of a small sample size is one of the main reasons. The presence of publication bias was assessed from the funnel plot of precision by log odds ratio method and statistically tested using Egger’s regression test.

## Results

### Case—Control study

We have analyzed MTHFR 677 C>T polymorphism in 588 patients and 508 controls (Table 2). There was no significant difference in the distribution of genotypes between cases and controls in north Indian (P = 0.932) or south Indian (P = 0.865) groups (Table 2). Statistical analysis using dominant, co-dominant, and recessive models also detected no significant association of c.677C>T polymorphism with breast cancer in north Indian or south Indian groups (Table 3).

**Table 2:** Genotypes distribution between cases and controls.

Population	Cases			Controls		
	CC	CT	TT	CC	CT	TT
**North Indian**	229	89	13	127	48	6
**South Indian**	208	45	4	259	63	5
**Total**	437	134	17	386	111	11

**Table 3:** Statistical comparison of genotypes distribution between cases and controls.

Comparisons	North Indian			South Indian			Pooled		
	OR	95%CI	P	OR	95%CI	P	OR	95%CI	P
**CC vs. (CT+TT)**	1.04	0.70–1.55	0.82	0.89	0.59–1.35	0.6	1.09	0.83–1.43	0.52
**CC vs. CT**	1.02	0.68–1.55	0.88	0.88	0.58–1.35	0.59	1.06	0.80–1.41	0.66
**CC vs. TT**	1.2	0.44–3.23	0.71	0.99	0.26–3.75	1	1.36	0.63–2.95	0.42
**TT vs. CT**	0.85	0.30–2.39	0.76	0.89	0.22–3.51	1	0.78	0.35–1.73	0.54
**(CC+CT)vs. TT**	1.19	0.44–3.19	0.72	1.01	0.27–3.83	1	1.34	0.62–2.89	0.44
**CC vs. CT vs. TT**	-	-	0.93	-	-	0.86	-	-	0.68

### Meta—Analysis

#### Literature search

**Fig 1:**
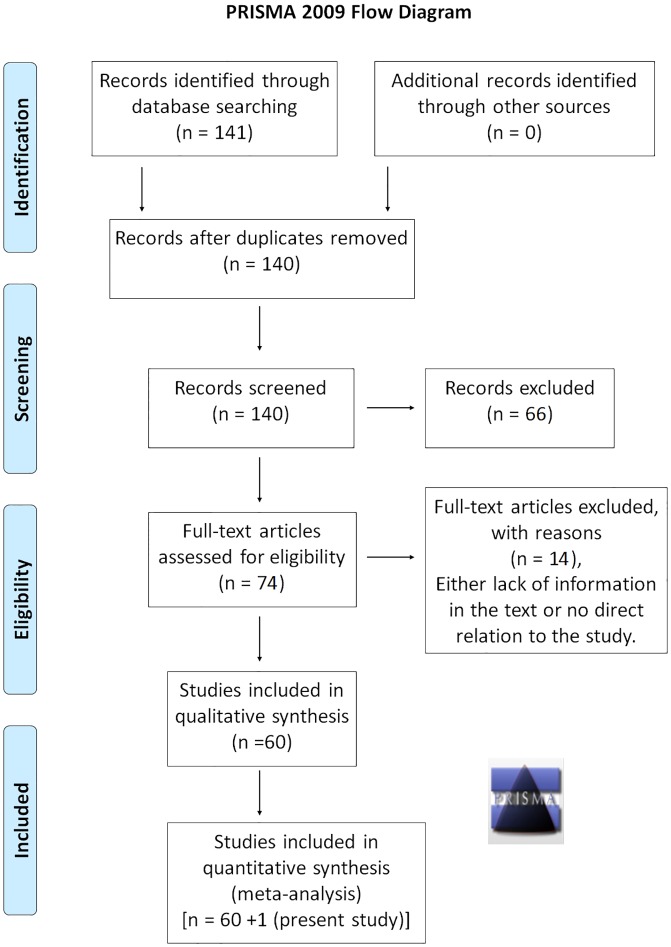
PRISMA flow diagram. The chart shows systematic review of the literature for inclusion/exclusion of the studies in meta-analysis.

A total of 141 studies were retrieved upon literature search. After removal of one duplicate [21], 140 records were screened for inclusion in the study. Seventy four of these were found to be relevant to our study on 677C>T substitution and breast cancer. Eight studies were excluded due to lack of direct relation to the 677C>T SNP and breast cancer [22, 23, 24, 25, 26, 27, 28, 29], while six others were excluded as they lacked information required for meta- analysis [30, 31, 32, 33, 34, 35]. Hence, a total of 60 studies [1, 3–7, 9–13, 36–84] following strict selection criteria were included in the meta-analysis. Along with our data from India, data for a total of 28031 cases and 31880 controls were included in the meta-analysis (Fig. 1). Genotype data for all the studies are tabulated in S1 Table.

#### Pooled analysis

**Fig 2:**
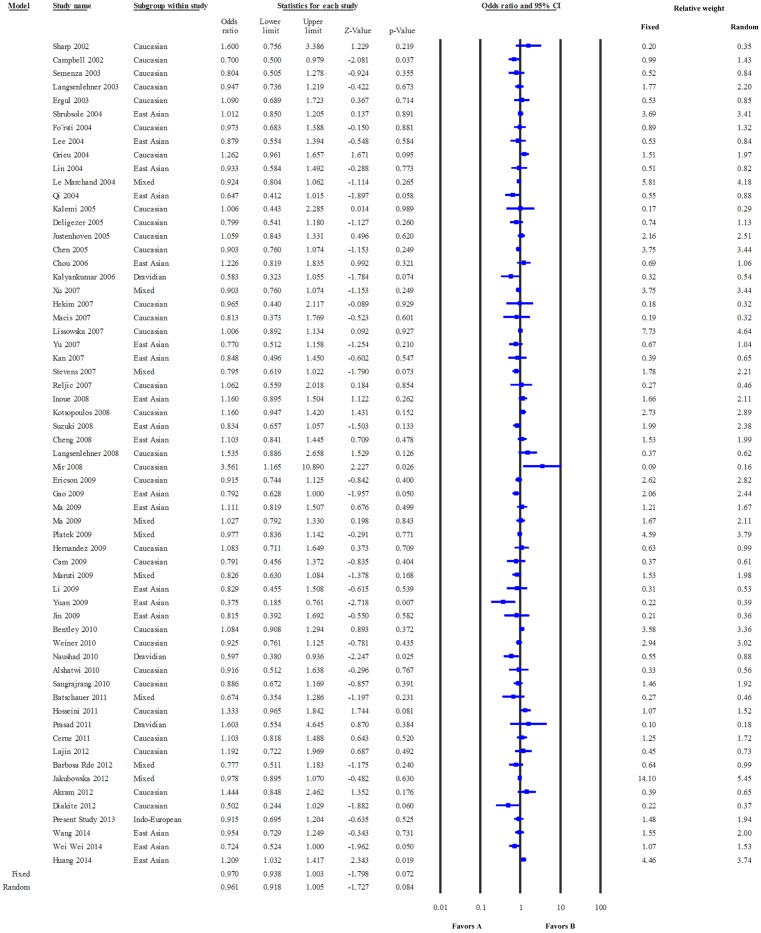
Meta-analysis. Forest plot on data pooled from all eligible studies. The Z value shows the degree and direction of relationship, wheras the P value shows the significance of the relationship. The horizontal bar shows the range of OR with a square in the centre, the size of which is directly proportional to the weight given to each study. The direction of projection of the horizontal bar shows the direction of association.

**Fig 3:**
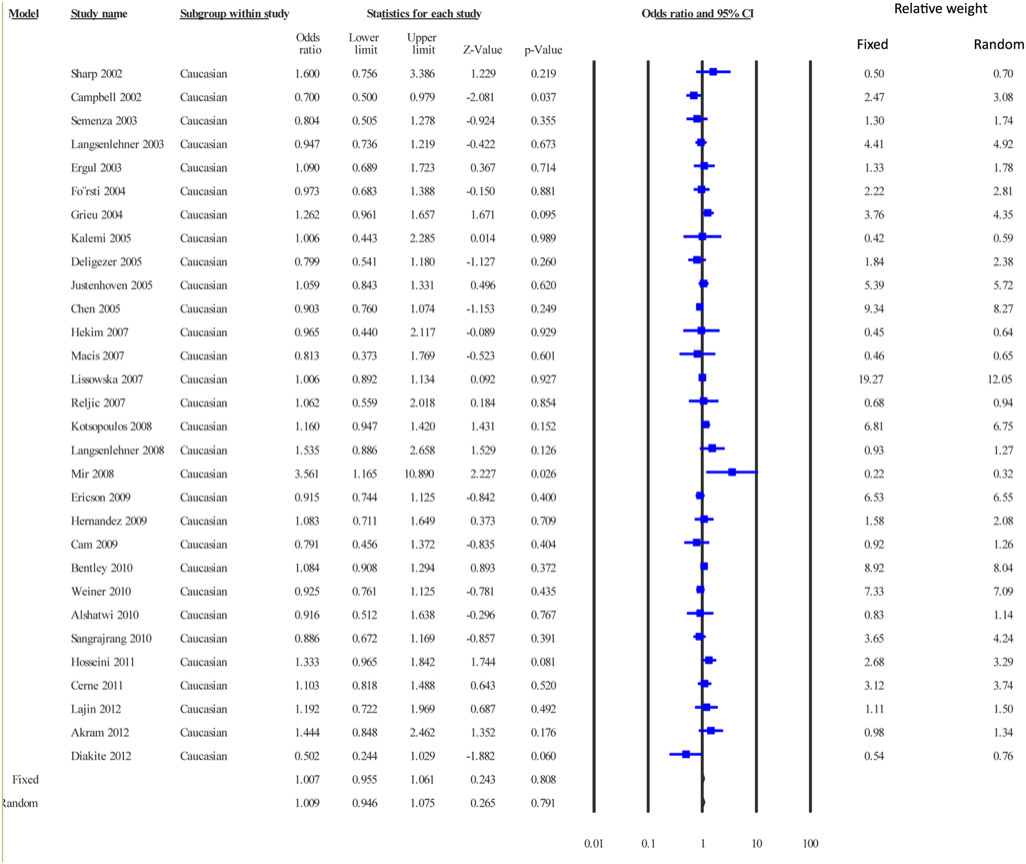
Meta-analysis. Forest plot on data pooled from studies on Caucasian populations. All other parameters are as detailed in Fig. 2.

**Fig 4:**
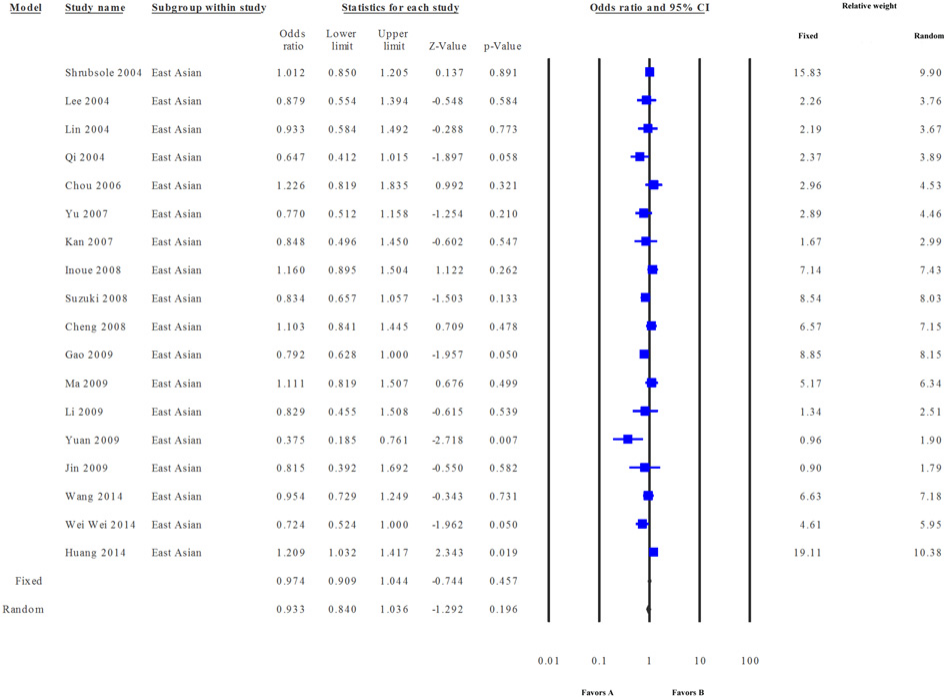
Meta-analysis. Forest plot on data pooled from studies on East Asian populations. All other parameters are as detailed in Fig. 2.

The pooled data showed a low level of heterogeneity based on the Thompson and Higgins classification index (P_Heterogeneity_ = 0.02, I^2^ = 29.55). Meta-analysis suggested no significant association between c.677 C>T and breast cancer risk in the dominant model (Fixed effect model: OR = 0.97, P = 0.072; Random effects model: OR = 0.96, P = 0.084, Fig. 2) or the recessive model (Fixed effect model: OR = 1.05, P = 0.089; Random effects model: OR = 1.08, P = 0.067, figure not shown). In the sub-group analysis, only dominant model was adopted. In the Caucasian group, the data were homogeneous (P_heterogeneity =_ 0.19, I^2^ = 17.96), and both Fixed effect (odds ratio = 1.007, P = 0.808) and Random effects models (odds ratio = 1.009, P = 0.791) suggested a lack of association between the study polymorphism and the disease risk (Fig. 3). Similarly, the data for East Asians (P_heterogeneity_ = 0.01, I^2^ = 47.21) showed low level of heterogeneity, and no correlation between c.677 C>T substitution and breast cancer risk was evident in this group (Fixed effects model: OR = 0.974, P = 0.457 and Random effects model: OR = 0.933, P = 0.196) (Fig. 4).

#### Sensitivity analysis based on sample size

To identify sensitive studies affecting the results of meta-analysis, thirteen studies based on small sample size (<100) in either of the study groups were excluded [4, 10, 42, 44, 47, 49, 54, 59, 67–69, 74, 81]. Re-analysis of the data showed more homogeneity (P_Heterogeneity_ = 0.06, I^2^ = 25.58), but the substitution did not correlate with breast cancer (fixed effect model: odds ratio = 0.975, P = 0.142; random effects model: odds ratio = 0.970, P = 0.174).

#### Publication bias

The distribution of studies on the funnel plot was almost symmetrical, suggesting the absence of publication bias in the overall analysis (S1 Fig.). This was further confirmed by Egger’s regression intercept test (P = 0.259). Similarly, a symmetrical distribution of studies on the funnel plot for the Caucasian population showed absence of bias that was confirmed by Egger’s regression intercept test (P = 0.555). But, the East Asian data also showed the presence of publication bias, confirmed by the Egger’s regression intercept test (P = 0.017).

## Discussion

In the present case-control study on 588 patients and 508 healthy controls, we found no association between *MTHFR* 677 C>T gene polymorphism and breast cancer amongst Indian women. Among other studies on Indian populations, Mir et al. showed that individuals carrying 677 C>T substitution had a 3.5 fold less risk of breast cancer (OR = 3.41, 95%CI = 3.1–3.7, P<0.02) in a north Indian Caucasian population [59]. On the other hand, Kalyankumar et al. (2006) and Prasad et al. (2011) reported a lack of association between MTHFR variants and the risk of breast cancer in south Indian populations [47, 75]. However, Naushad et al. (2010) suggested that the c.677C>T substitution is an independent risk factor for breast cancer in Indian women of Dravidian ethnicity (OR = 1.74, 95% CI = 1.11–2.73) [72]. The authors suggested that the risk is related to thermolabile MTHFR enzyme that has the tendency to lose its active dimer form with a reduction in the FAD- binding capacity and loss in specific activity. The same contrast in the results of case-control studies is seen in other studies on diverse ethnicities; however, a relatively large number of studies support lack of association between 677 C>T substitution and breast cancer risk. Among studies on Chinese populations, only two out of 13 showed association of 677 C>T substitution with breast cancer risk, whereas all others stated no such correlation. Ten out of 30 studies on Caucasians reported an association between 677 C>T substitution and breast cancer risk, while others stated lack of such a correlation.

Meta-analysis is a powerful tool to reach consensus on heterogeneous data reported across studies. At-least seven meta-analyses have been conducted to pool genotype data in order to reach a consensus. However, interestingly, even meta-analysis on the relation of 677 C>T substitution with breast cancer has been equally heterogeneous with respect to the analysis models, stringency, and the outcomes. Zintzaras (2006) compared CC versus TT genotypes in a meta-analysis on `18 studies (5476 cases and 7336 controls) and found that 677C>T polymorphism and breast cancer are very closely associated with each other in pre-menopausal women [15]. Macis et al. (2007) investigated the relationship by pooling data from 18 case-control studies and found that 677C>T is strongly associated with breast cancer in both dominant and recessive genetic models [10]. Supporting the conclusions further, Zhang et al. (2010), Qi et al. (2010), and Liang et al (2013) conducted meta-analyses on 37 studies (15260 cases and 20411 controls), 41 studies (16480 cases and 22388 controls), and 22 studies (6103 cases and 7913 controls), respectively, and reported significant association in comparison of CC versus TT and in recessive model [16–18]. Rai (2014) and Li et al (2014) conducted meta-analyses on 36 studies (8040 cases and 10008 controls) and 57 studies (25877 cases and 29781 controls), respectively, and found a significant association across all genetic models in Asian population [85, 86]. Interestingly, all the above described meta-analyses suggested that c.677C>T polymorphism is a risk factor for breast cancer.

We undertook a meta-analysis on data pooled from all eligible studies that fitted a strictly defined inclusion and exclusion criteria. The present meta-analysis pooled data for 28031 cases and 31880 controls from sixty one studies. Our results suggest that *MTHFR* 677C>T polymorphism is not associated with the risk of breast cancer in either dominant (P = 0.084) or recessive genetic model (P = 0.067). It must be appreciated that pooling small studies into the meta-analysis resolves the issues related to sample size, but the biasness introduced by inappropriate representation of population-wise genotypes ratio would fail to correct. Therefore, we conducted a sensitivity analysis after excluding studies using a sample size smaller than 100 in either of the case/control groups. Interestingly, we failed to detect an association between 677 C>T substitution and breast cancer, suggesting robustness of the method used in pooled analysis. Lack of publication bias further suggests that the results have not been influenced by any missing study. Analysis ethnicity-wise was considered so as to uncover the association in a particular ethnic population. A majority of the published studies were conducted on Caucasian and East-Asian populations with a small number on other populations. Therefore, we undertook two sub-analysis on Caucasian and East-Asian data; however, lack of a correlation between 677 C>T substitution and breast cancer risk was consistent. It is interesting to note the reported association of this substitution with breast cancer in a number of case-control studies; however, it has not been suggested what could be the mechanism leading to cancer in relatively poor one carbon metabolism as the nutritional deficiencies have not been reported to directly raise cancer risk.

In conclusion, while a majority of the case-control studies deny an association between the 677 C>T polymorphism and breast cancer, meta-analyses till date have consistently supported existence of an association. Our stringent statistical approach and thorough sensitivity analyses have suggested that 677C>T does not affect breast cancer risk. About 50% of the studies pooled in this analysis had been undertaken on populations of Caucasian ethnicity and 30% on East Asian populations. Therefore, our results would be more relevant to the populations of these ethnicities and caution must be ensured while extrapolating them to other populations.

## Supporting Information

S1 Table Studies included in the meta-analysis. All the studies that were included in the meta-analysis have been listed with details of the observed genotypes.
(DOCX)

S1 Fig Publication bias. Funnel plot of precision by log odds ratio. Each empty dot represents one study included in the analysis and each solid dot represents one imputed study.
(TIF)

## References

[1] Sangrajrang S, Sato S, Sakamoto H, Ohnami S, Khuhaprema T, Yoshida T. Genetic polymorphism in folate and alcohol metabolism and breast cancer risk: a case- control study in Thai women. Breast Cancer Res Treat. 2010; 123: 885–893. 10.1007/s10549-010-0804-4 20180013

[2] Yu L, Chen J. Association of MHTFR Ala222Val (rs1801133) polymorphism and breast cancer susceptibility: An update meta-analysis based on 51 research studies. Diagn Pathol. 2012; 7: 171 10.1186/1746-1596-7-171 23217001 PMC3536596

[3] Shrubsole MJ, Gao YT, Cai O, Shu XO, Dai Q, Hebert JR, et al *MTHFR* Polymorphisms, Dietary Folate Intake, and Breast Cancer Risk: Results from the Shanghai Breast Cancer Study. Cancer Epidemiol Biomarkers Prev. 2004; 13:190–196. 14973091 10.1158/1055-9965.epi-03-0273

[4] Sharp L, Little J, Schofield AC, Pavlidou E, Cotton SC, Meidzybrodzka Z, et al Folate and breast cancer: the role of polymorphisms in methylenetetrahydrofolate reductase (MTHFR).Cancer Lett. 2002; 181:65–71. 12430180 10.1016/s0304-3835(02)00030-7

[5] Ericson U, Sonestedt E, Ivarsson M.I, Gullberg B, Carlson J, Olsson H, et al Folate intake, methylenetetrahydrofolate reductase polymorphisms, and breast cancer risk in women from the Malmö Diet and Cancer cohort. Cancer Epidemiol Biomarkers Prev. 2009; 18:1101–1110. 10.1158/1055-9965.EPI-08-0401 19336565

[6] Kotsopoulos J, Zhang WW, Zhang S, McCready D, Trudeau M, Zhang P, et al Polymorphisms in folate metabolizing enzymes and transport proteins and the risk of breast cancer. Breast Cancer Res Treat. 2008; 112:585–593. 10.1007/s10549-008-9895-6 18204969

[7] Chou YC, Wu MH, Yu JC, Lee MS, Yang T. Genetic polymorphisms of the methylenetetrahydrofolate reductase gene, plasma folate levels and breast cancer susceptibility: a case-control study in Taiwan. Carcinogenesis. 2006; 27: 2295–2300. 16777985 10.1093/carcin/bgl108

[8] Langevin SM, Lin D, Matsuo K, Gao CM, Takezaki T, Stolzenberg-Solomom RZ, et al Review and pooled analysis of studies on MTHFR C677T polymorphism and esophageal cancer. Toxicol Lett. 2009; 184:73–80. 10.1016/j.toxlet.2008.09.003 18840514 PMC3512563

[9] Lee SA, Kang D, Nishio H, Lee MJ, Kim DH, Han W, et al Methylenetetrahydrofolate reductase polymorphism, diet, and breast cancer in Korean women. Exp Mol Med. 2004; 36:116–121. 15150439 10.1038/emm.2004.17

[10] Macis D, Maisonneuve P, Johansson H, Bonanni B, Botteri E, Iodice S, et al Methylenetetrahydrofolate reductase (MTHFR) and breast cancer risk: a nested-case-control study and a pooled meta-analysis. Breast Cancer Res Treat. 2007; 106:263–271. 17260091 10.1007/s10549-006-9491-6

[11] Hosseini M, Houshmand M, Ebrahimi A. MTHFR polymorphisms and breast cancer risk. Arch Med Sci. 2011; 7:134–137. 10.5114/aoms.2011.20618 22291746 PMC3258688

[12] Deligezer U, Akisik EE, Dalay N. Homozygosity at the C677T of the MTHFR gene is associated with increased breast cancer risk in the Turkish population. In Vivo. 2005; 19:889–893. 16097444

[13] Le Marchand L, Haiman CA, Wilkens LR, Kolonel LN, Henderson BE. MTHFR polymorphisms, diet, HRT, and breast cancer risk: the multiethnic cohort study.Cancer Epidemiol Biomarkers Prev. 2004; 13:2071–2077. 15598763

[14] Lewis SJ, Harbord RM, Harris R, Smith GD. Meta-analyses of Observational and Genetic Association Studies of Folate Intakes or Levels and Breast Cancer Risk. Journal of the National Cancer Institute. 2006; 98: 122. 10.1093/jnci/djj440 17105984

[15] Zintzaras E. Methylenetetrahydrofolate reductase gene and susceptibility to breast cancer: a meta-analysis. Clin Genet. 2006; 69: 327–336. 16630166 10.1111/j.1399-0004.2006.00605.x

[16] Zhang J, Qiu LX, Wang ZH, Wu XH, Liu XJ. MTHFR C677T polymorphism associated with breast cancer susceptibility: a meta-analysis involving 15,260 cases and 20,411 controls. Breast Cancer Res Treat. 2010; 123: 549–555. 10.1007/s10549-010-0783-5 20143151

[17] Qi X, Ma X, Yang X, Fan L, Zhang Y. Methylenetetrahydrofolate reductase polymorphisms and breast cancer risk: a meta-analysis from 41 studies with 16,480 cases and 22,388 controls. Breast Cancer Res Treat. 2011; 123: 499–506. 10.1007/s10549-010-0773-7 20135343

[18] Liang H, Yan Y, Li T, Li R, Li M, Li S, et al Methylenetetrahydrofolate reductase polymorphisms and breast cancer risk in Chinese population: a meta-analysis of 22 case-control studies. Tumour Biol. 2013; 35: 1695–1701. 10.1007/s13277-013-1234-9 PMC3932174 24078451

[19] Huedo-Medina TB, Sanchez-Meca J, Marin-Martinez F, Botella J. Assessing heterogeneity in Meta- analysis: Q statistic or I2 Index. Physcol Methods. 2006; 11: 193–206. 10.1037/1082-989X.11.2.193 16784338

[20] Petitti DB. Approaches to heterogeneity in meta-analysis. Stat Med. 2001; 20: 3625–3633. 11746342 10.1002/sim.1091

[21] Mohammad NS, Yedluri R, Addepalli P, Gottumukkala SR, Digumarti RR, Kutala VK. Aberrations in one-carbon metabolism induce oxidative DNA damage in sporadic breast cancer. Mol Cell Biochem. 2011; 349:159–167. 10.1007/s11010-010-0670-8 21113649

[22] Bailey LB. Folate, methyl-related nutrients, alcohol, and the MTHFR 677C—>T polymorphism affect cancer risk: intake recommendations. J Nutr. 2003; 133: 3748S–3753S. 14608109 10.1093/jn/133.11.3748S

[23] Sohn KJ, Jang H, Campan M, Weisenberger DJ, Dickhout J, Wang YC, et al The methylenetetrahydrofolate reductase C677T mutation induces cell-specific changes in genomic DNA methylation and uracil misincorporation: a possible molecular basis for the site-specific cancer risk modification. Int J Cancer. 2009; 124:1999–2005. 10.1002/ijc.24003 19123462 PMC2692263

[24] Ferroni P, Palmirotta R, Martini F, Riondino S, Savonarola A, Spila A, et al Determinants of homocysteine levels in colorectal and breast cancer patients. Anticancer Res. 2009; 29:4131–4138. 19846961

[25] Eroğlu A, Akar N. Factor V Leiden, prothrombin G20210A and methylenetetrahydrofolate reductase (MTHFR) C677T polymorphisms and the risk of tamoxifen-associated thromboembolism in breast cancer patients. Thromb Res. 2011; 127:384–385. 10.1016/j.thromres.2010.10.025 21093891

[26] Tamura T, Kurata M, Kondo T, Goto Y, Kamiya Y, Kawai S, et al Preventive medical services not covered by public health insurance at Daiko Medical Center in Japan, 2004–2011. Nagoya J Med Sci. 2012; 74:115–121. 22515117 PMC4831256

[27] Akilzhanova A, Nurkina Z, Momynaliev K, Ramanculov E, Zhumadilov Z. Genetic profile and determinants of homocysteine levels in Kazakhstan patients with breast cancer. Anticancer Res. 2013; 33: 4049–4059. 24023349

[28] Iwasaki M, Mizusawa J, Kasuga Y, Yokoyama S, Onuma H, Nishimura H, et al Green Tea Consumption and Breast Cancer Risk in Japanese Women: A Case-Control Study. Nutr Cancer. 2014; 66: 57–67. 10.1080/01635581.2014.847963 24274352

[29] Ozen F, Erdis E, Sik E, Silan F, Uludag A, Ozdemir O. Germ-line MTHFR C677T, FV H1299R and PAI-1 5G/4G variations in breast carcinoma. Asian Pac J Cancer Prev. 2013; 14:2903–2908. 23803051 10.7314/apjcp.2013.14.5.2903

[30] Martin YN, Olson JE, Ingle JN, Vierkant RA, Fredericksen ZS. Methylenetetrahydrofolate reductase haplotype tag single-nucleotide polymorphisms and risk of breast cancer. Cancer Epidemiol Biomarkers Prev. 2006; 15: 2322–2324. 17119067 10.1158/1055-9965.EPI-06-0318

[31] Huang MY, Wang YH, Chen FM, Lee SC, Fang WY, Cheng TL, et al Multiple Genetic Polymorphisms of GSTP1 313AG, MDR1 3435CC, and MTHFR 677CC highly correlated with early relapse of breast cancer patients in Taiwan. Ann Surg Oncol. 2008; 15:872–880. 18095031 10.1245/s10434-007-9719-7

[32] Tao MH, Shields PG, Nie J, Marian C, Ambrosone CB, McCann SE, et al DNA promoter methylation in breast tumors: no association with genetic polymorphisms in MTHFR and MTR. Cancer Epidemiol Biomarkers Prev. 2009; 18:998–1002. 10.1158/1055-9965.EPI-08-0916 19240236 PMC3837294

[33] Knechtel G, Hofmann G, Gerger A, Renner W, Langsenlehner T, Skandera J, et al Analysis of common germline polymorphisms as prognostic factors in patients with lymph node-positive breast cancer. J Cancer Res Clin Oncol. 2010; 136:1813–1819. 10.1007/s00432-010-0839-2 20204402 PMC11828221

[34] Papandreou CN, Doxani C, Zdoukopoulos N, Vlachostergios PJ, Hatzidaki E, Bakolas G, et al Evidence of association between methylenetetrahydrofolate reductase gene and susceptibility to breast cancer: a candidate-gene association study in a South-eastern European population. DNA Cell Biol. 2012; 31:193–198. 10.1089/dna.2011.1292 21875371

[35] Eroglu A, Karabiyik A, Akar N. The association of protease activated receptor 1 gene-506 I/D polymorphism with disease-free survival in breast cancer patients. Ann Surg Oncol. 2012; 19: 1365–1369. 10.1245/s10434-011-1969-8 21822552

[36] Campbell IG, Baxter SW, Eccles DM, Choong DY. Methylenetetrahydrofolate reductase polymorphism and susceptibility to breast cancer. Breast Cancer Res. 2002; 4:R14 12473175 10.1186/bcr457 PMC137931

[37] Semenza JC, Delfino RJ, Ziogas A, Anton-Culver H. Breast cancer risk and methylenetetrahydrofolate reductase polymorphism. Breast Cancer Res Treat. 2003; 77:217–223. 12602921 10.1023/a:1021843019755

[38] Langsenlehner U, Krippl P, Renner W, Yazdani-Biuki B, Wolf G. The common 677C>T gene polymorphism of methylenetetrahydrofolate reductase gene is not associated with breast cancer risk. Breast Cancer Res Treat. 2003; 81: 169–172. 14572159 10.1023/A:1025752420309

[39] Ergul E, Sazci A, Utkan Z, Canturk NZ. Polymorphisms in the MTHFR gene are associated with breast cancer. Tumour Biol. 2003; 24: 286–290. 15004488 10.1159/000076460

[40] Forsti A, Angelini S, Festa F, Sanyal S, Zhang Z, Grzybowska E, et al Single nucleotide polymorphisms in breast cancer. Oncol Rep. 2004; 11:917–922. 15010895

[41] Grieu F, Powell B, Beilby J, Iacopetta B. Methylenetetrahydrofolate reductase and thymidylate synthase polymorphisms are not associated with breast cancer risk or phenotype. Anticancer Res. 2004; 24: 3215–3219. 15510613

[42] Lin WY, Chou YC, Wu MH, Huang HB, Jeng YL. The MTHFR C677T polymorphism, estrogen exposure and breast cancer risk: a nested case-control study in Taiwan. Anticancer Res. 2004; 24: 3863–3868. 15736423

[43] Qi J, Miao XP, Tan W, Yu CY, Liang G, Lu WF, Lin DX. Association between genetic polymorphisms in methylenetetrahydrofolate reductase and risk of breast cancer. Chin J Oncol. 2004; 26:287–289 15312365

[44] Kalemi TG, Lambropoulos AF, Gueorguiev M, Chrisafi S, Papazisis KT. The association of p53 mutations and p53 codon 72, Her 2 codon 655 and MTHFR C677T polymorphisms with breast cancer in Northern Greece. Cancer Lett. 2005; 222: 57–65. 15837541 10.1016/j.canlet.2004.11.025

[45] Justenhoven C, Hamann U, Pierl CB, Rabstein S, Pesch B. One-carbon metabolism and breast cancer risk: no association of MTHFR, MTR, and TYMS polymorphisms in the GENICA study from Germany. Cancer Epidemiol Biomarkers Prev. 2005; 14:3015–3018. 16365030 10.1158/1055-9965.EPI-05-0592

[46] Chen J, Gammon MD, Chan W, Palomeque C, Wetmur JG. One-carbon metabolism, MTHFR polymorphisms, and risk of breast cancer. Cancer Res. 2005; 65: 1606–1614. 15735051 10.1158/0008-5472.CAN-04-2630

[47] Kalyankumar C, Jamil K. Methylenetetrahydrofolate Reductase (*MTHFR*) C677T and A1298C Polymorphisms and Breast Cancer in South Indian Population. International Journal of Cancer Research. 2006; 2: 143–151.

[48] Xu X, Gammon MD, Zhang H, Wetmur JG, Rao M. Polymorphisms of one-carbon-metabolizing genes and risk of breast cancer in a population-based study. Carcinogenesis. 2007; 28: 1504–1509. 17372271 10.1093/carcin/bgm061

[49] Hekim N, Ergen A, Yaylim I, Yilmaz H, Zeybek U, Ozturk O, et al No association between methylenetetrahydrofolate reductase C677T polymorphism and breast cancer. Cell Biochem Funct. 2007; 25:115–117. 16134079 10.1002/cbf.1274

[50] Lissowska J, Gaudet MM, Brinton LA, Chanock SJ, Peplonska B. Genetic polymorphisms in the one-carbon metabolism pathway and breast cancer risk: a population-based case-control study and meta-analyses. Int J Cancer. 2007; 120: 2696–2703. 17311260 10.1002/ijc.22604

[51] Yu CP, Wu MH, Chou YC, Yang T, You SL, Chen CJ, Sun CA. Breast cancer risk associated with multigenotypic polymorphisms in folate-metabolizing genes: a nested case-control study in Taiwan. Anticancer Res. 2007; 27:1727–1732. 17595805

[52] Kan XX, Zou TN, Wu XY, Wang X. Association between MTHFR genotype polymorphism and breast cancer susceptibility in human population from Yunnan. Cancer Res Prev Treat. 2007; 34:716–718.

[53] Stevens VL, McCullough ML, Pavluck AL, Talbot JT, Feigelson HS, Thun MJ, et al Association of polymorphisms in one-carbon metabolism genes and postmenopausal breast cancer incidence. Cancer Epidemiol Biomarkers Prev. 2007; 16:1140–1147. 17548676 10.1158/1055-9965.EPI-06-1037

[54] Reljic A, Simundic AM, Topic E, Nikolac N, Justinic D. The methylenetetrahydrofolate reductase (MTHFR) C677T polymorphism and cancer risk: the Croatian case-control study. Clin Biochem. 2007; 40: 981–985. 17573062 10.1016/j.clinbiochem.2007.05.005

[55] Inoue M, Robien K, Wang R, Van Den Berg DJ, Koh WP, et al Green tea intake, MTHFR/TYMS genotype and breast cancer risk: the Singapore Chinese Health Study. Carcinogenesis. 2008; 29:1967–1972. 10.1093/carcin/bgn177 18669903 PMC2574755

[56] Suzuki T, Matsuo K, Hirose K, Hiraki A, Kawase T, Watanabe M, et al One-carbon metabolism- related gene polymorphisms and risk of breast cancer. Carcinogenesis. 2008; 2:356–362. 10.1093/carcin/bgm295 18174236

[57] Cheng CW, Yu JC, Huang CS, Shieh JC, Fu YP. Polymorphism of cytosolic serine hydroxymethyltransferase, estrogen and breast cancer risk among Chinese women in Taiwan. Breast Cancer Res Treat. 2008; 111: 145–155. 17896178 10.1007/s10549-007-9754-x

[58] Langsenlehner T, Renner W, Yazdani-Biuki B, Langsenlehner U. Methylenetetrahydrofolate reductase (MTHFR) and breast cancer risk: a nested-case-control study and a pooled meta-analysis. Breast Cancer Res Treat. 2008; 107: 459–460. 17453338 10.1007/s10549-007-9564-1

[59] Mir MM, Dar JA, Dar NA, Dar MS, Salam I. Combined impact of polymorphism of folate metabolism genes; glutamate carboxypeptidase, methylene tetrahydrofolate reductase and methionine synthase reductase on breast cancer susceptibility in Kashmiri women. Int J Health Sci. 2008; 2: 3–14. PMC3068715 21475466

[60] Gao CM, Tang JH, Cao HX, Ding JH, Wu JZ, Wang J et al MTHFR polymorphisms, dietary folate intake and breast cancer risk in Chinese women. J Hum Genet. 2009; 54:414–418. 10.1038/jhg.2009.57 19557016

[61] Ma E, Iwasaki M, Kobayashi M, Kasuga Y, Yokoyama S. Dietary intake of folate, vitamin B2, vitamin B6, vitamin B12, genetic polymorphism of related enzymes, and risk of breast cancer: a case-control study in Japan. Nutr Cancer. 2009; 61: 447–456. 10.1080/01635580802610123 19838916

[62] Platek ME, Shields PG, Marian C, McCann SE, Bonner MR. Alcohol consumption and genetic variation in methylenetetrahydrofolate reductase and 5-methyltetrahydrofolate-homocysteine methyltransferase in relation to breast cancer risk. Cancer Epidemiol Biomarkers Prev. 2009; 18: 2453–2459. 10.1158/1055-9965.EPI-09-0159 19706843 PMC2941988

[63] Henriquez-Hernandez LA, Murias-Rosales A, Hernandez Gonzalez A, Cabrera De Leon A, Diaz-Chico BN. Gene polymorphisms in TYMS, MTHFR, p53 and MDR1 as risk factors for breast cancer: a case-control study. Oncol Rep. 2009; 22: 1425–1433. 19885596 10.3892/or_00000584

[64] Cam R, Eroglu A, Egin Y, Akar N. Dihydrofolate reductase (DHRF) 19-bp intron-1 deletion and methylenetetrahydrofolate reductase (MTHFR) C677T polymorphisms in breast cancer. Breast Cancer Res Treat. 2009; 115: 431–432. 10.1007/s10549-008-0054-x 18498051

[65] Maruti SS, Ulrich CM, Jupe ER, White E. MTHFR C677T and postmenopausal breast cancer risk by intakes of one-carbon metabolism nutrients: a nested case-control study. Breast Cancer Res. 2009; 11: R9 10.1186/bcr2225 20030812 PMC2815555

[66] Ma E, Iwasaki M, Junko I, Hamada GS, Nishimoto IN. Dietary intake of folate, vitamin B6, and vitamin B12, genetic polymorphism of related enzymes, and risk of breast cancer: a case-control study in Brazilian women. BMC Cancer. 2009; 9: 122 10.1186/1471-2407-9-122 19389261 PMC2684745

[67] Li WD, Chen SQ. Association of methylenetetrahydrofolate reductase C677T polymorphism and breast cancer risk. J Prac Med. 2009; 25:2031–2033.

[68] Yuan H, Xu XY, Wang ZL. The relation between polymorphisms of methylenetetrahydrofolate reductase C677T and the risk of breast cancer. J MuDanJiang Med Univ. 2009; 30:2–4.

[69] Jin ZZ, Lu Q, Ge DH, Zong M, Zhu QH. Effect of the methylenetetrahydrofolate reductase gene C677Tpolymorphism on C-erbB-2 methylation status and its association with cancer. Mol Med Rep. 2009; 2:283–289. 10.3892/mmr_00000097 21475826

[70] Bentley AR, Raiszadeh F, Stover PJ, Hunter DJ, Hankinson SE. No association between cSHMT genotypes and the risk of breast cancer in the Nurses' Health Study. Eur J Clin Nutr. 2010; 64: 108–110. 10.1038/ejcn.2009.104 19707223 PMC3033771

[71] Vainer AS, Boiarskikh UA, Voronina EN, Selezneva IA, Sinkina TV. Polymorphic variants of folate metabolizing genes (C677T and A1298C MTHFR, C1420T SHMT1 and G1958A MTHFD) are not associated with the risk of breast cancer in West Siberian Region of Russia. Mol Biol (Mosk). 2010; 44: 816–823. 21090237

[72] Naushad SM, Pavani A, Digumarti RR, Gottumukkala SR, Kutala VK. Epistatic interactions between loci of one-carbon metabolism modulate susceptibility to breast cancer. Mol Biol Rep. 2010; 38: 4893–4901. 10.1007/s11033-010-0631-z 21161404

[73] Alshatwi AA. Breast cancer risk, dietary intake, and methylenetetrahydrofolate reductase (MTHFR) single nucleotide polymorphisms. Food Chem Toxicol. 2010; 48: 1881–1885. 10.1016/j.fct.2010.04.028 20417243

[74] Batschauer AP, Cruz NG, Oliveira VC, Coelho FF, Santos IR. HFE, MTHFR, and FGFR4 genes polymorphisms and breast cancer in Brazilian women. Mol Cell Biochem. 2011; 357: 247–253. 10.1007/s11010-011-0895-1 21625954

[75] Prasad VV, Wilkhoo H. Association of the functional polymorphism C677T in the methylenetetrahydrofolate reductase gene with colorectal, thyroid, breast, ovarian, and cervical cancers. Onkologie. 2011; 34:422–426. 10.1159/000331131 21934341

[76] Ziva Cerne J, Stegel V, Gersak K, Novakovic S. Lack of association between methylenetetrahydrofolate reductase genetic polymorphisms and postmenopausal breast cancer risk. Mol Med Rep. 2012; 4: 175–179. 10.3892/mmr.2010.406 21461582

[77] Lajin B, Alhaj Sakur A, Ghabreau L, Alachkar A. Association of polymorphisms in one-carbon metabolizing genes with breast cancer risk in Syrian women. Tumour Biol. 2012; 33: 1133–1139. 10.1007/s13277-012-0354-y 22373582

[78] Carvalho Barbosa Rde C, Menezes DC, Freire TF, Sales DC, Alencar VH. Associations of polymorphisms of folate cycle enzymes and risk of breast cancer in a Brazilian population are age dependent. Mol Biol Rep. 2012; 39: 4899–4907. 10.1007/s11033-011-1285-1 22134752

[79] Jakubowska A, Rozkrut D, Antoniou A, Hamann U, Scott RJ, McGuffog L, et al Association of PHB 1630 C>T and MTHFR 677 C>T polymorphisms with breast and ovarian cancer risk in BRCA1/2 mutation carriers: results from a multicenter study. Br J Cancer. 2012; 106:2016–2024. 10.1038/bjc.2012.160 22669161 PMC3388557

[80] Akram M, Malik FA, Kayani MA. Mutational analysis of the MTHFR gene in breast cancer patients of Pakistani population. Asian Pac J Cancer Prev. 2012; 13:1599–1603. 22799374 10.7314/apjcp.2012.13.4.1599

[81] Diakite B, Tazzite A, Hamzi K, Jouhadi H, Nadifi S. Methylenetetrahydrofolate reductase C677T polymorphism and breast cancer risk in Moroccan women. Afr Health Sci. 2012; 12: 204–209. 10.4314/ahs.v12i2.20 23056029 PMC3462529

[82] Wang ZG, Cui W, Yang LF, Zhu YQ, Wei WH. Association of dietary intake of folate and MTHFR genotype with breast cancer risk. Genet Mol Res. 2014; 13:5446–5451. 10.4238/2014.July.24.24 25078601

[83] Weiwei Z, Liping C, Dequan L. Association between dietary intake of folate, vitamin B6, B12 & MTHFR, MTR Genotype and breast cancer risk. Pak J Med Sci. 2014; 30:106–1010. 10.12669/pjms.301.4189 24639841 PMC3955552

[84] Huang CY, Chang WS, Shui HA, Hsieh YH, Loh CH, Wang HC, et al Evaluation of the contribution of Methylenetetrahydrofolate Reductase genotypes to Taiwan Breast Cancer. Anticancer Res. 2014; 34:4109–4115. 25075036

[85] Rai V. The Methylenetetrahydrofolate Reductase C677T Polymorphism and Breast Cancer Risk in Asian Populations. Asian Pac J Cancer Prev. 2014; 15:5853–5860. 25081713 10.7314/apjcp.2014.15.14.5853

[86] Li K, Li W, Dong X. Association of 677 C>T (rs1801133) and 1298 A>C (rs1801131) polymorphisms in the MTHFR gene and breast cancer susceptibility: a meta-analysis based on 57 individual studies. PLoS One. 2014; 9:e71290 10.1371/journal.pone.0071290 24945727 PMC4063741

