# Supplementary material for: MTHFR 677C>T Polymorphism and the Risk of Breast Cancer: Evidence from an Original Study and Pooled Data for 28031 Cases and 31880 Controls
Source: PLoS One. 2015 Mar 24;10(3):e0120654. doi: 10.1371/journal.pone.0120654 (PMC4372432; doi:10.1371/journal.pone.0120654)
Supplement: S1 Table — All the studies that were included in the meta-analysis have been listed with details of the observed genotypes. (DOCX) [file pone.0120654.s001.docx]

S1_Table.

| **No.** | **First author** | **Year** | **Country** | **Ethnicity** | **Source of controls** | **Cases** | | | |  |  | **Controls** | | | |  |  | **HWE** |
| --- | --- | --- | --- | --- | --- | --- | --- | --- | --- | --- | --- | --- | --- | --- | --- | --- | --- | --- |
|  |  |  |  |  |  |  |  |  |  |  |  |  |  |  |  |  |  |  |
|  |  |  |  |  |  | **CC** | **CT** | **TT** | **CT+TT** | **CT+CC** | **Total** | **CC** | **CT** | **TT** | **CT+TT** | **CT+CC** | **Total** | **P** |
|  |  |  |  |  |  |  | | | |  |  |  | | | |  |  |  |
| 1 | Sharp 2002 | 2002 | UK | Caucasian | Population-based | 30 | 19 | 5 | 24 | 49 | 54 | 25 | 21 | 11 | 32 | 46 | 57 | 0.1032 |
| 2 | Campbell 2002 | 2002 | UK | Caucasian | Hospital-based | 140 | 162 | 33 | 195 | 302 | 335 | 118 | 92 | 23 | 115 | 210 | 233 | 0.4199 |
| 3 | Semenza 2003 | 2003 | USA | Caucasian | Hospital-based | 42 | 58 | 5 | 63 | 100 | 105 | 112 | 111 | 24 | 135 | 223 | 247 | 0.6434 |
| 4 | Langsenlehner 2003 | 2003 | Austria | Caucasian | Population-based | 208 | 222 | 64 | 286 | 430 | 494 | 215 | 215 | 65 | 280 | 430 | 495 | 0.3334 |
| 5 | Ergul 2003 | 2003 | Turkey | Caucasian | Hospital-based | 60 | 41 | 17 | 58 | 101 | 118 | 94 | 87 | 12 | 99 | 181 | 193 | 0.1641 |
| 6 | Shrubsole 2004 | 2004 | China | East Asian | Population-based | 374 | 555 | 183 | 738 | 929 | 1112 | 387 | 577 | 196 | 773 | 964 | 1160 | 0.4425 |
| 7 | Fo¨rsti 2004 | 2004 | Finland | Caucasian | Not stated | 134 | 81 | 8 | 89 | 215 | 223 | 181 | 104 | 13 | 117 | 285 | 298 | 0.6891 |
| 8 | Lee 2004 | 2004 | Korea | East Asian | Hospital-based | 58 | 96 | 32 | 128 | 154 | 186 | 50 | 80 | 17 | 97 | 130 | 147 | 0.0763 |
| 9 | Grieu 2004 | 2004 | Australia | Caucasian | Population-based | 166 | 141 | 27 | 168 | 307 | 334 | 242 | 259 | 50 | 309 | 501 | 551 | 0.1002 |
| 10 | Lin 2004 | 2004 | China | East Asian | Population-based | 43 | 38 | 7 | 45 | 81 | 88 | 173 | 145 | 24 | 169 | 318 | 342 | 0.3887 |
| 11 | Le Marchand 2004 | 2004 | USA | Mixed | Population-based | 573 | 479 | 137 | 616 | 1052 | 1189 | 1211 | 920 | 283 | 1203 | 2131 | 2414 | 0 |
| 12 | Qi 2004 | 2004 | China | East Asian | Population-based | 42 | 104 | 71 | 175 | 146 | 217 | 59 | 105 | 54 | 159 | 164 | 218 | 0.5931 |
| 13 | Kalemi 2005 | 2005 | Greece | Caucasian | Not stated | 19 | 16 | 7 | 23 | 35 | 42 | 23 | 20 | 8 | 28 | 43 | 51 | 0.3125 |
| 14 | Deligezer 2005 | 2005 | Turkey | Caucasian | Not stated | 98 | 68 | 23 | 91 | 166 | 189 | 128 | 83 | 12 | 95 | 211 | 223 | 0.7591 |
| 15 | Justenhoven 2005 | 2005 | Germany | Caucasian | Population-based | 249 | 274 | 61 | 335 | 523 | 584 | 261 | 279 | 93 | 372 | 540 | 633 | 0.1935 |
| 16 | Chen 2005 | 2005 | New york | Caucasian | Population-based | 398 | 476 | 189 | 665 | 874 | 1063 | 440 | 509 | 155 | 664 | 949 | 1104 | 0.6886 |
| 17 | Chou 2006 | 2006 | China | East Asian | Hospital-based | 73 | 51 | 18 | 69 | 124 | 142 | 132 | 120 | 33 | 153 | 252 | 285 | 0.4748 |
| 18 | Kalyankumar 2006 | 2006 | India | Dravidian | Hospital-based | 45 | 37 | 6 | 43 | 82 | 88 | 61 | 31 | 3 | 34 | 92 | 95 | 0.6934 |
| 19 | Xu 2007 | 2007 | USA | Mixed | Population-based | 398 | 476 | 189 | 665 | 874 | 1063 | 440 | 509 | 155 | 664 | 949 | 1104 | 0.6886 |
| 20 | Hekim 2007 | 2007 | Turkey | Caucasian | Not stated | 22 | 16 | 2 | 18 | 38 | 40 | 38 | 26 | 4 | 30 | 64 | 68 | 0.8715 |
| 21 | Macis 2007 | 2007 | Italy | Caucasian | Population-based | 14 | 20 | 12 | 32 | 34 | 46 | 28 | 41 | 11 | 52 | 69 | 80 | 0.5111 |
| 22 | Lissowska 2007 | 2007 | Poland | Caucasian | Population-based | 982 | 815 | 177 | 992 | 1797 | 1974 | 1132 | 915 | 235 | 1150 | 2047 | 2282 | 0.0138 |
| 23 | Yu 2007 | 2007 | China | East Asian | Population-based | 56 | 54 | 9 | 63 | 110 | 119 | 225 | 170 | 25 | 195 | 395 | 420 | 0.3363 |
| 24 | Kan 2007 | 2007 | China | East Asian | Population-based | 74 | 29 | 22 | 51 | 103 | 125 | 65 | 29 | 9 | 38 | 94 | 103 | 0.0418 |
| 25 | Stevens 2007 | 2007 | USA | Mixed | Population-based | 208 | 224 | 62 | 286 | 432 | 494 | 236 | 193 | 65 | 258 | 429 | 494 | 0.0126 |
| 26 | Reljic 2007 | 2007 | Croatia | Caucasian | Population-based | 40 | 44 | 9 | 53 | 84 | 93 | 27 | 34 | 4 | 38 | 61 | 65 | 0.1143 |
| 27 | Inoue 2008 | 2008 | Singapore | East Asian | Population-based | 239 | 120 | 21 | 141 | 359 | 380 | 393 | 226 | 43 | 269 | 619 | 662 | 0.1782 |
| 28 | Kotsopoulos 2008 | 2008 | Canada | Caucasian | Hospital-based | 383 | 421 | 140 | 561 | 804 | 944 | 252 | 341 | 87 | 428 | 593 | 680 | 0.0867 |
| 29 | Suzuki 2008 | 2008 | Japan | East Asian | Hospital-based | 150 | 220 | 84 | 304 | 370 | 454 | 338 | 425 | 146 | 571 | 763 | 909 | 0.5219 |
| 30 | Cheng 2008 | 2008 | China | East Asian | Hospital-based | 185 | 133 | 31 | 164 | 318 | 349 | 268 | 221 | 41 | 262 | 489 | 530 | 0.6236 |
| 31 | Langsenlehner 2008 | 2008 | Austria | Caucasian | Not stated | 51 | 43 | 11 | 54 | 94 | 105 | 40 | 48 | 17 | 65 | 88 | 105 | 0.6846 |
| 32 | Mir 2008 | 2008 | India | Caucasian | Hospital-based | 29 | 6 | 0 | 6 | 35 | 35 | 19 | 12 | 2 | 14 | 31 | 33 | 0.9542 |
| 33 | Ericson 2009 | 2009 | Sweden | Caucasian | Population-based | 255 | 235 | 50 | 285 | 490 | 540 | 531 | 452 | 91 | 543 | 983 | 1074 | 0.7067 |
| 34 | Gao 2009 | 2009 | China | East Asian | Population-based | 202 | 305 | 117 | 422 | 507 | 624 | 235 | 301 | 88 | 389 | 536 | 624 | 0.5924 |
| 35 | Ma 2009 | 2009 | Japan | East Asian | Hospital-based | 124 | 183 | 81 | 264 | 307 | 388 | 115 | 188 | 84 | 272 | 303 | 387 | 0.663 |
| 36 | Platek 2009 | 2009 | USA | Mixed | Population-based | 429 | 446 | 119 | 565 | 875 | 994 | 788 | 795 | 219 | 1014 | 1583 | 1802 | 0.3975 |
| 37 | Hernandez 2009 | 2009 | Spain | Caucasian | Population-based | 52 | 65 | 18 | 83 | 117 | 135 | 107 | 138 | 47 | 185 | 245 | 292 | 0.8225 |
| 38 | Cam 2009 | 2009 | Turkey | Caucasian | Not stated | 48 | 49 | 13 | 62 | 97 | 110 | 47 | 42 | 6 | 48 | 89 | 95 | 0.3986 |
| 39 | Maruti 2009 | 2009 | USA | Mixed | Population-based | 133 | 139 | 46 | 185 | 272 | 318 | 301 | 284 | 62 | 346 | 585 | 647 | 0.6725 |
| 40 | Ma 2009 | 2009 | Brazil | Mixed | Hospital-based | 225 | 188 | 45 | 233 | 413 | 458 | 222 | 187 | 49 | 236 | 409 | 458 | 0.3093 |
| 41 | Li 2009 | 2009 | China | East Asian | Population-based | 38 | 17 | 10 | 27 | 55 | 65 | 90 | 50 | 3 | 53 | 140 | 143 | 0.1874 |
| 42 | Yuan 2009 | 2009 | China | East Asian | Hospital-based | 16 | 35 | 29 | 64 | 51 | 80 | 32 | 35 | 13 | 48 | 67 | 80 | 0.5156 |
| 43 | Jin 2009 | 2009 | China | East Asian | Not stated | 18 | 20 | 3 | 23 | 38 | 41 | 49 | 41 | 10 | 51 | 90 | 100 | 0.7421 |
| 44 | Bentley 2010 | 2010 | USA | Caucasian | Hospital-based | 346 | 402 | 191 | 593 | 748 | 939 | 429 | 592 | 205 | 797 | 1021 | 1226 | 0.9747 |
| 45 | Vainer 2010 | 2010 | Russia | Caucasian | Population-based | 399 | 364 | 74 | 438 | 763 | 837 | 386 | 326 | 66 | 392 | 712 | 778 | 0.8084 |
| 46 | Naushad 2010 | 2010 | India | Dravidian | Hospital-based | 185 | 56 | 3 | 59 | 241 | 244 | 205 | 39 | 0 | 39 | 244 | 244 | 0.1748 |
| 47 | Alshatwi 2010 | 2010 | saudi arabia | Caucasian | Hospital-based | 34 | 50 | 16 | 66 | 84 | 100 | 36 | 49 | 15 | 64 | 85 | 100 | 0.8009 |
| 48 | Sangrajrang 2010 | 2010 | Thailand | Caucasian | Hospital-based | 410 | 144 | 9 | 153 | 554 | 563 | 366 | 110 | 11 | 121 | 476 | 487 | 0.4266 |
| 49 | Batschauer 2011 | 2011 | Brazil | Mixed | Population-based | 27 | 34 | 7 | 41 | 61 | 68 | 42 | 34 | 9 | 43 | 76 | 85 | 0.5927 |
| 50 | Hosseini 2011 | 2011 | Tehran | Caucasian | Hospital-based | 168 | 84 | 42 | 126 | 252 | 294 | 150 | 90 | 60 | 150 | 240 | 300 | 0 |
| 51 | Prasad 2011 | 2011 | India | Dravidian | Population-based | 124 | 5 | 1 | 6 | 129 | 130 | 116 | 8 | 1 | 9 | 124 | 125 | 0.0624 |
| 52 | Cerne 2011 | 2011 | Solvenia | Caucasian | Population-based | 222 | 238 | 62 | 300 | 460 | 522 | 108 | 124 | 37 | 161 | 232 | 269 | 0.8822 |
| 53 | Lajin 2012 | 2012 | Arab | Caucasian | Hospital-based | 60 | 47 | 12 | 59 | 107 | 119 | 58 | 58 | 10 | 68 | 116 | 126 | 0.3879 |
| 54 | [Barbosa Rde 2012](http://www.ncbi.nlm.nih.gov/pubmed?term=%22Carvalho%20Barbosa%20Rde%20C%22%5BAuthor%5D) | 2012 | Brazil | Mixed | Hospital-based | 76 | 83 | 17 | 100 | 159 | 176 | 87 | 70 | 19 | 89 | 157 | 176 | 0.3888 |
| 55 | Jakubowska 2012 | 2012 | Poland | Mixed | Population-based | 2032 | 2166 | 580 | 2746 | 4198 | 4778 | 1447 | 1481 | 422 | 1913 | 2928 | 3350 | 0.1562 |
| 56 | Akram 2012 | 2012 | Pakistan | Caucasian | Hospital-based | 65 | 25 | 20 | 45 | 90 | 110 | 55 | 45 | 10 | 55 | 100 | 110 | 0.8554 |
| 57 | Diakite 2012 | 2012 | Morocco | Caucasian | Hospital-based | 24 | 34 | 4 | 38 | 58 | 62 | 34 | 21 | 6 | 27 | 55 | 40 | 0.3187 |
| 58 | Present Study 2013 | 2013 | India | Indo-European | Hospital-based | 437 | 134 | 17 | 151 | 571 | 588 | 386 | 111 | 11 | 122 | 497 | 508 | 0.3708 |
| 59 | Wang 2014 | 2014 | China | East Asian | Hospital-based | 250 | 153 | 32 | 185 | 403 | 435 | 255 | 150 | 30 | 180 | 405 | 435 | 0.2228 |
| 60 | Wei Wei 2014 | 2014 | China | East Asian | Hospital-based | 156 | 97 | 44 | 141 | 253 | 297 | 185 | 93 | 28 | 121 | 278 | 306 | 0.0022 |
| 61 | Huang 2014 | 2014 | Taiwan | East Asian | Hospital-based | 596 | 533 | 103 | 636 | 1129 | 1232 | 538 | 519 | 175 | 694 | 1057 | 1232 | 0.0066 |
|  |  |  |  |  |  |  |  |  |  |  | 28031 |  |  |  |  |  | 31880 |  |
