# Supplementary figures and images for: MTHFR 677C>T Polymorphism and the Risk of Breast Cancer: Evidence from an Original Study and Pooled Data for 28031 Cases and 31880 Controls
Source: PLoS One. 2015 Mar 24;10(3):e0120654. doi: 10.1371/journal.pone.0120654 (PMC4372432; doi:10.1371/journal.pone.0120654)

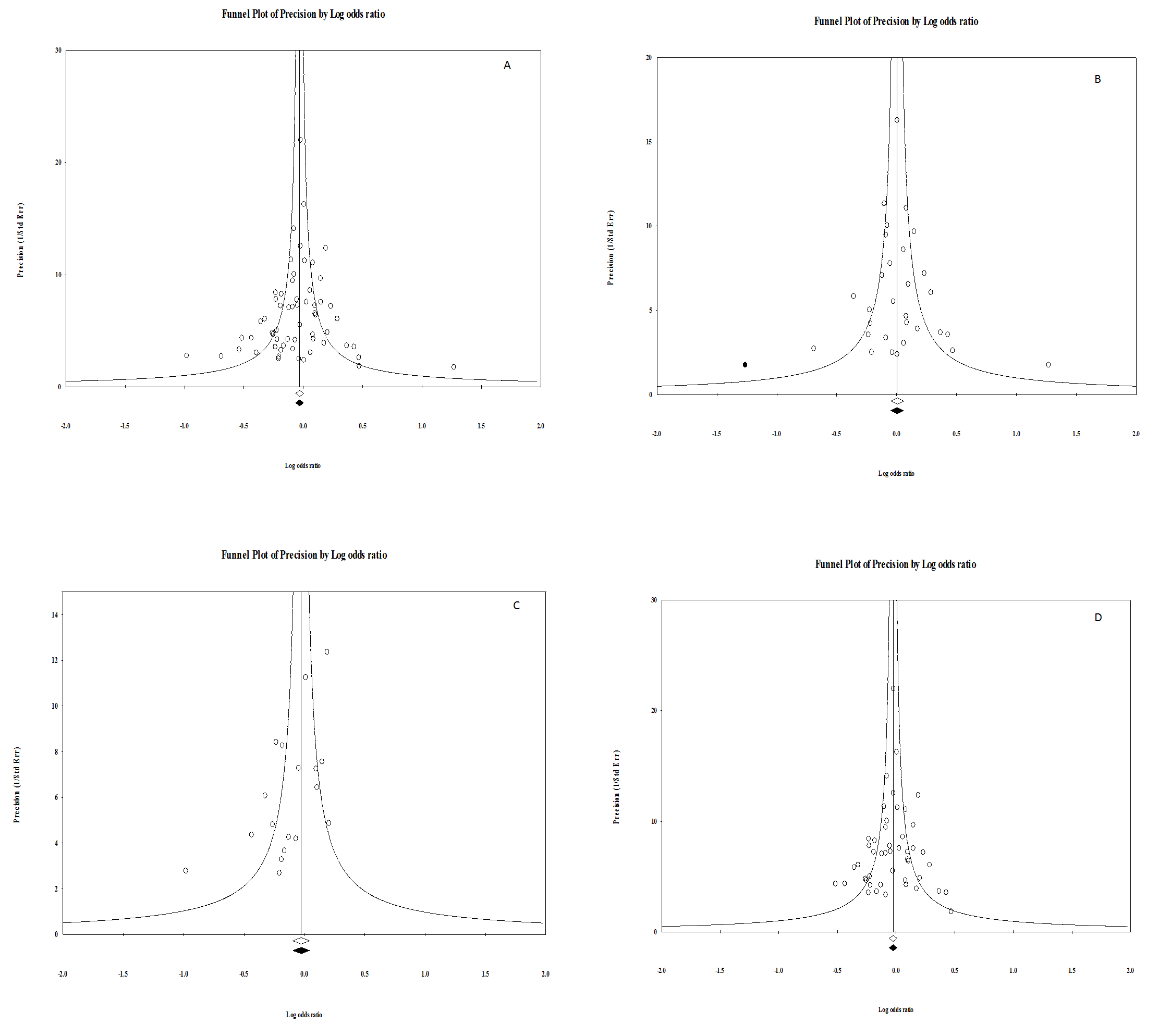

Supplement: S1 Fig — Funnel plot of precision by log odds ratio. Each empty dot represents one study included in the analysis and each solid dot represents one imputed study. (TIF) [file pone.0120654.s002.tif]
